# Effect of Toll-Like Receptor 4 on Synovial Injury of Temporomandibular Joint in Rats Caused by Occlusal Interference

**DOI:** 10.1155/2016/7694921

**Published:** 2016-06-20

**Authors:** Jingjing Kong, Yingying Yang, Shuzhen Sun, Jianli Xie, Xuefen Lin, Ping Ji

**Affiliations:** ^1^Key Laboratory of Oral Biomedicine of Shandong Province, Stomatological Hospital of Shandong University, Number 44, Wen Hua Xi Lu, Shandong Province, Jinan 250012, China; ^2^Jinan Stomatological Hospital, Number 101, Jing Liu Lu, Shandong Province, Jinan 250001, China; ^3^Ningbo Medical Treatment Center Lihuili Hospital, Ningbo, Zhejiang 315000, China

## Abstract

Synovitis is an important disease that causes intractable pain in TMJ. Some investigations suggested that the increasing expression of IL-1*β* secreted by synovial lining cells plays an important role in synovial inflammation and cartilage destruction in TMJ. In our previous research, the results demonstrated that TLR4 is involved in the expression of IL-1*β* in SFs from TMJ with lipopolysaccharide stimulation. However, the inflammatory response that occurred in synovial membrane is not caused by bacterial infection. In the current study, we investigated whether or not TLR4 participates in the inflammatory responses and the expression of IL-1*β* in synovial membrane of rats induced by occlusal interference. The results showed that obvious inflammation changes were observed in the synovial membranes and the expression of TLR4 and IL-1*β* was increased at both mRNA and protein levels in the occlusal interference rats. In addition, the inflammation reactions and the increased expression of IL-1*β* could be restrained by treatment with TAK-242, a blocker of TLR4 signaling. The results prompted us that the activation of TLR4 may be involved in the inflammatory reactions and increased expression of IL-1*β* in patients with synovitis and participate in the mechanisms of the initiation and development of synovial injury by regulating the expression of inflammatory mediators like IL-1*β* in synovial membranes.

## 1. Introduction

Temporomandibular disorder (TMD) is one of common and frequently occurring diseases in department of stomatology. A survey found that 9.7% of the population suffers from conditions covered by TMD group I diagnosis (myofascial pain) and 11.4% from conditions covered by TMD group II a diagnosis (disk displacement with reduction) [1]. The patients can experience some symptoms that seriously affect human normal life and work, for example, pain in Temporomandibular Joint (TMJ) and masticatory muscle and limited mouth opening. The mechanisms of the initiation and development of this disease are complicated and not completely clear, and a lot of etiologic factors may be attributed to the onset of disorder, as biomedical and psychological as well as psychosocial impact factors and occlusal interferences [2–4].

Synovitis is an inflammation mainly occurs in synovial membrane and joint capsule of TMJ. A series of investigations [5, 6] on TMD has revealed the occurrence of inflammation in the synovial membrane. Various inflammatory mediators are thought to be involved in joint pathology, including interleukin- (IL-) 1*β*, tumor necrosis factor- (TNF-) *α*, and matrix metalloproteinases (MMPs) [7–9]. IL-1*β* was reported to be expressed by synovial lining cells and endothelial cells of blood vessels [10], and it is suggested that the increasing expression of IL-1*β* plays an important role in synovial inflammation and cartilage destruction [11, 12].

In our previous research, we found that treatment with lipopolysaccharide (LPS) could increase Toll-like receptor (TLR) 4 (a transmembrane protein) and IL-1*β* expression at both mRNA and protein levels in synovial fibroblasts (SFs) separated from TMJ of rat, and the increased expression of IL-1*β* could be blocked by treatment with TAK-242, a blocker of TLR4 signaling, and the cell surface receptor TLR4 is involved in the expression of IL-1*β* in SFs from TMJ with LPS stimulation [13]. However, the inflammatory response that occurs in synovial membrane is not caused by bacterial infection as we all know. In the current study, we created an occlusal interference animal model [14] to induce synovial injury by bonding crowns with a thickness of 0.6 mm to the right mandibular first molars of rats and describe inflammatory response in the synovial membranes and expression of TLR4 and IL-1*β*. Besides, we injected TAK-242 into upper compartment of TMJ to describe the change of TLR4 and IL-1*β* expression in synovial lining cells, and we wanted to investigate whether or not TLR4 participates in the inflammatory responses and the expression of IL-1*β* in rats induced by occlusal interference.

## 2. Materials and Methods

### 2.1. Subjects

Thirty-six male wistar rats (6-week old, obtained from the Shandong University Center of Laboratory Animals, China) were housed under a 12-h light/dark cycle with food and water available ad libitum. This study was approved by the Animal Care and Use Committee at the Shandong University.

### 2.2. Animal Model of Occlusal Interference

Rats were anesthetized with intraperitoneal injection of pentobarbital sodium (0.5%, 40 mg/kg). A metallic crown (0.6 mm, uniform thickness) was bonded to the right mandibular first molar using resin cement (Super-Bond C&B, Osaka, Japan). Crowns were fabricated using cobalt chromium casting alloys and designed to cover the occlusal, buccal, lingual, and medial surfaces of the molars. Sham-treated rats in the control group were anesthetized and their mouths were forced opened for approximately five minutes using a protocol similar to the occlusal interference groups; however, no crowns were cemented.

Thirty-six rats were randomly divided into three groups (twelve rats in each group) and treated as follows: (1) control group, these rats were anesthetized and mouths were forced open for about 5 min and received saline injections (10 *μ*L, twice a week) into upper joint cavities of both sides of TMJs, (2) occlusal interference group, these rats were treated to create an occlusal interference animal model according to methods above and received saline injections (10 *μ*L, twice a week) into upper joint cavities of both sides of TMJs, (3) TAK-242 group, these rats were treated to create an occlusal interference animal model according to methods above and received TAK-242 injections (3 mg/kg [15], diluted in 10 *μ*L DMSO, twice a week; Invitrogen, San Diego, CA, USA) into upper joint cavities of both sides of TMJs.

### 2.3. Tissue Preparation

After two weeks, six rats in each group were randomly selected and were euthanized by overdose pentobarbital sodium. Then, rats were perfused with heparinized saline followed by a cold fixative containing 4% paraformaldehyde in 0.01 M phosphate buffer saline (PBS, pH 7.2). The right TMJs were removed, fixed in 4% paraformaldehyde, and then demineralized in 15% EDTA. After decalcification in 10% EDTA, the TMJs were dehydrated, embedded in paraffin, and sectioned on the sagittal plane at a thickness of 4 *μ*m.

The other six rats in each group were also anesthetized with overdose pentobarbital sodium. The synovial tissues were harvested from the right TMJs, rinsed with cold sterile saline solution, and stored at −80°C for real-time quantitative polymerase chain reaction (PCR) assay.

### 2.4. Histopathologic Examination

The sagittal sections of the central portion of the rat TMJ were selected from each TMJ in all rats and stained with hematoxylin and eosin. The histopathological findings were evaluated using measure that is described as follows [16, 17]:

1. Synovial lining hyperplasia was graded on a scale from 0 to 2: grade 0, staining of 1–3 layers; grade 1, staining of 4–6 layers; and grade 2, staining of 7 or more layers.
2. Dilated vasculature was graded on a scale from 0 to 3: grade 0, not present; grade 1, involving less than one-third of the synovial membrane length; grade 2, involving one-third to two-thirds of the synovial membrane length; grade 3, involving more than two-thirds of the synovial membrane length.
3. Fibrin deposits were graded on a scale from 0 to 3 (as described for the vasculature).
4. Vascularity was graded on a scale from 0 to 2: grade 0, a limited number (less than 5) of blood vessel profiles/mm^2^; grade 1, focal occurrence of 5–10 small blood vessel profiles/mm^2^; grade 2, focal occurrence of a large number (more than 10) of small blood vessel profiles/mm^2^.

### 2.5. Immunohistochemistry

After routine deparaffinization and rehydration, the sections underwent antigen retrieval in 0.125% trypsin-EDTA (Solarbio, Beijing, China) for 20 min at 37°C. Histostain*™*-Plus kits (ZSGB-Bio, Beijing, China) were used according to the manufacturer's recommendations. After incubation in goat serum, sections were incubated with the primary antibodies against TLR4 and IL-1*β* (1 : 1000, Cell Signaling, Beverly, MA, USA), respectively, overnight at 4°C. After rinsing with 0.01 M PBS, the sections were exposed to goat anti-rabbit secondary antibody (ZSGB-Bio, Beijing, China) for 30 min at 37°C, then were exposed to a solution of horseradish peroxidase-conjugated avidin-biotin complex (ZSGB-Bio, Beijing, China) for 20 min at 37°C. Then, sections were visualized with 0.1% 3, 30-diaminobenzidine dihydrochloride (DAB) (ZSGB-Bio, Beijing, China), and the sections were counterstained with hematoxylin. The digital images were captured using a microscopy digital camera system (Olympus, Tokyo, Japan). The results were evaluated semiquantitatively using the Image-Pro Plus 6.0 software. Five sections per rat were assayed in high power, and the mean optical density (MOD) was measured, respectively. The mean of MOD of five sections was seen as relative protein expression of this rat.

### 2.6. Real-Time Quantitative PCR

Total RNA was extracted from synovial tissues using Trizol (Invitrogen, Carlsbad, CA, USA) according to the manufacturer's protocol. The first strand complementary DNA (cDNA) was synthesized by reverse transcription using SYBR Prime Script TM RT reagent Kit (Takara, Dalian, China). The levels of target mRNA in synovial tissues were analyzed by quantitative real-time PCR using SYBR Green I dye (Takara, Dalian, China). The primer pairs used for PCR were as follows: forward 5′-CCTGTGCAATTTGACCATTG-3′ and reverse 5′- AAGCATTCCCACCTTTGTTG-3′ for TLR4, forward 5′-ACAAGGAGAGACAAGCAACGA-3′ and reverse 5′-TCTGCTTGAGAGGTGCTGATG-3′ for IL-1*β*, and forward 5′-GAAGGTGAAGGTCGGAGTCG-3′ and reverse 5′-GAAGATGGTGATGGGATTTC-3′ for glyceraldehyde-3-phosphate dehydrogenase (GAPDH).

The amplification was performed in triplicate on a LightCycler 480 QPCR System (Roche Diagnostics Ltd., Bern, Switzerland). Each gene was normalized against the corresponding GAPDH levels and relative gene expression of each sample was fold change (2^−ΔΔCt^) using the control group as calibrator.

### 2.7. Statistical Analysis

Normally distributed variables were expressed as means ± SD. Unpaired Student's *t*-test was used to compare differences between groups. Differences in data values were defined significant at a *P* < 0.05 using SPSS statistical software package Version 17.0.

## 3. Results

### 3.1. Histological Examination

**Figure 1:**
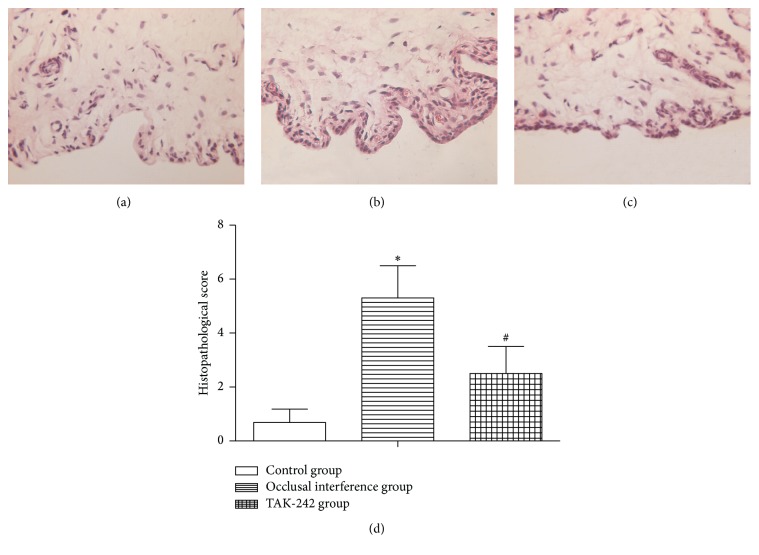
Histological examination of synovial membranes. (a) Control group. (b) Occlusal interference group. (c) TAK-242 group. (d) The histopathological score of each group. As the results shown, in comparison with the control group, the histopathological score was significantly increased in the occlusal interference group. However, this effect could be inhibited significantly after treatment with the TAK-242. Data shows all the values from independent samples of *n* = 6, ^*∗*^ *P* < 0.05 versus control group and ^#^ *P* < 0.05 versus occlusal interference group.

In the control group (Figure 1(a)), the synovial membranes of the TMJs did not show inflammatory changes. In the occlusal interference group (Figure 1(b)), obvious inflammation changes were observed in the synovial membranes, such as apparent hyperplasia of synovial lining cells, dilated blood vessels, proliferation of blood vessels, and fibrin deposition. As shown in Figure 1(d), in comparison with that in the controls, the histopathological score was significantly increased in the occlusal interference group. In the TAK-242 group (Figure 1(c)), the treatment of TAK-242 markedly inhibited the inflammatory reactions, although slight hyperplasia of synovial lining cells and dilated blood vessels were still present. As shown in Figure 1(d), the histopathological score became significantly lower after treatment with the TAK-242 when compared with the occlusal interference group.

### 3.2. The Expression of TLR4 in the Synovial Membranes

**Figure 2:**
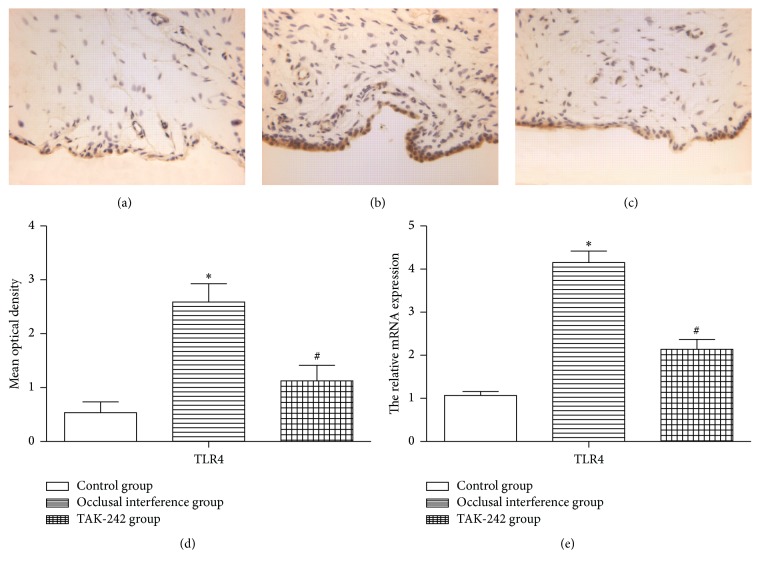
The expression of TLR4 in the synovial membranes. (a) Immunohistochemical staining for TLR4 in the membranes of the control group. (b) Immunohistochemical staining for TLR4 in the membranes of occlusal interference group. (c) Immunohistochemical staining for TLR4 in the membranes of TAK-242 group. (d) The mean optical density of each group. (e) The relative mRNA expression of TLR4 of each group. As the results shown, in comparison with the control group, the expression of TLR4 was significantly increased in the occlusal interference group at both protein and mRNA levels. However, this effect could be inhibited significantly after treatment with the TAK-242. Data shows all the values from independent samples of *n* = 6, ^*∗*^ *P* < 0.05 versus control group and ^#^ *P* < 0.05 versus occlusal interference group.

As shown in Figure 2(a), the immunohistochemistry revealed that there are few synovial membranes could be stained, and the synovial membranes in the control group hardly expressed TLR4. Compared with the control group, the area of synovial membranes stained was increased by the experiment of occlusal interference (Figure 2(b)), and the expression of TLR4 (Figure 2(d)) in the occlusal interference group was improved. The same result was also found in the mRNA expression (Figure 2(e)). In the TAK-242 group, treatment with TAK-242 could reduce the area of synovial membranes stained (Figure 2(c)). As shown in Figure 2(d), the occlusal interference induced increased expression of TLR4 was significantly reduced by the injection of TAK-242 compared with the occlusal interference group. Consistent with the protein change, the mRNA expression of TLR4 was also reduced (Figure 2(e)).

### 3.3. Effect of TLR4 on the Expression of IL-1*β* in the Synovial Membranes

**Figure 3:**
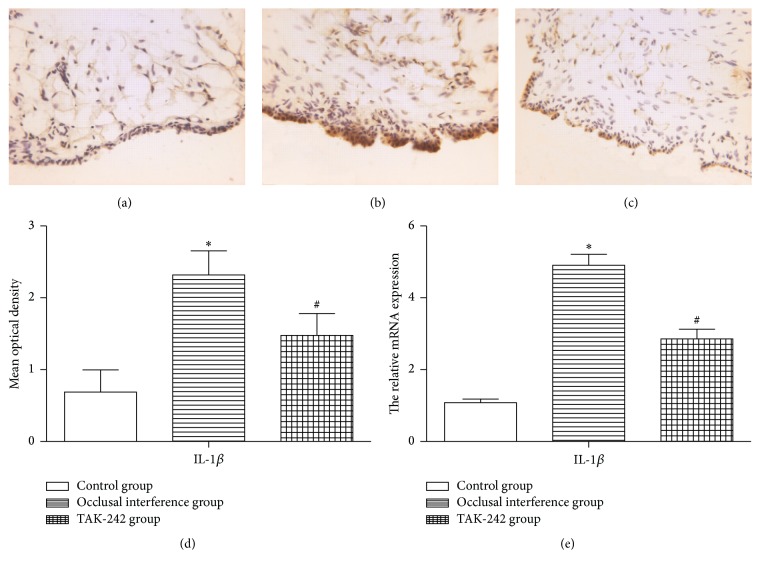
Effect of TLR4 on the expression of IL-1*β* in the synovial membranes. (a) Immunohistochemical staining for IL-1*β* in the membranes of the control group. (b) Immunohistochemical staining for IL-1*β* in the membranes of occlusal interference group. (c) Immunohistochemical staining for IL-1*β* in the membranes of TAK-242 group. (d) The mean optical density of each group. (e) The relative mRNA expression of IL-1*β* of each group. As the results shown, in comparison with the control group, the expression of IL-1*β* was significantly increased in the occlusal interference group at both protein and mRNA levels. However, this effect could be inhibited significantly after treatment with the TAK-242. Data shows all the values from independent samples of *n* = 6, ^*∗*^ *P* < 0.05 versus control group and ^#^ *P* < 0.05 versus occlusal interference group.

The results of immunohistochemistry staining for IL-1*β* in the synovial membranes of the TMJ in each groups were shown in Figures 3(a), 3(b), and 3(c). Compared with the control group, the expression of IL-1*β* of synovial membranes in the occlusal interference group was improved at both protein (Figure 3(d)) and mRNA (Figure 3(e)) levels. In the occlusal interference group, the treatment with TAK-242 significantly reduced occlusal interference-enhanced IL-1*β* expression at both protein (Figure 3(d)) and mRNA (Figure 3(e)) levels compared with the occlusal interference group.

## 4. Discussion

The patients of synovitis often suffered from pain in TMJ, and pain was the main reason that prompts patients to seek treatment at the hospital. The disease will continue to progress if patients do not receive effective treatment. Occlusion was defined as the balanced relationship between the incising or masticating surfaces of the maxillary and mandibular teeth. Experimental occlusal interference in animals could result in mandibular condyle bone remodeling [18], and another study [19] showed changes in blood flow in TMJ induced by experimental occlusal interference, and these changes possibly related to tissue damage and inflammation in TMJ. Occlusal interference was a tooth contact that inhibits the remaining occluding surfaces from achieving stable and harmonious contacts and changed the stress in articular cavity. The synovial membrane was sensitive tissue that feels stress in the articular cavity, and may occurred pathologic changes. In this study, we created an occlusal interference animal model by bonding crowns with a thickness of 0.6 mm to right mandibular first molar and observed obvious inflammation changes in the synovial membranes, such as apparent hyperplasia of synovial lining cells, dilated blood vessels, proliferation of blood vessels, and fibrin deposition. We induced synovial injury successfully by this method and provided experimental basis for the following research.

TLR4 is a member of the TLR (Toll-like receptor) family of transmembrane proteins, recognizes conserved pathogen associated molecular patterns like lipopolysaccharide (LPS), viral double-stranded RNA, bacterial flagella, and viral and bacterial CpG DNA, and generates innate immune responses to pathogens by activating a cascade of proinflammatory events [20]. Recent studies have found that endogenous ligands such as saturated free fatty acids [21] and high mobility group box-1 protein [22] can also activate TLR4. When a ligand binds to TLR4 and its coreceptors CD14 and MD-2, the adaptor molecules are recruited to the Toll/IL-1 receptor (TIR) domain of TLR4. This interaction cascade enables downstream signaling and mediates activation of a transcriptional factor and nuclear factor- (NF-) *κ*B, resulting in induction of proinflammatory genes, such as those encoding TNF-*α*, IL-6, and IL-1*β* [23, 24]. A serious of studies has demonstrated that the TLR4 signaling pathways play an important role in the progression of many diseases by mediating the expression of proinflammatory cytokines. Edfeldt et al. suggested that hyporesponsive TLR4 polymorphisms affect the susceptibility to myocardial infarction in men and that TLR4-mediated innate immunity plays a role in the pathogenesis of myocardial infarction [25]. A report identified that the interaction TLR4 signaling pathway is involved in the development of lung ischemia reperfusion injury (LIRI) [26]. Kim et al. cultivated the cartilage cells isolated from patients with osteoarthritis and detected increased expression of TLR4 mRNA [27]. In our previous study, we found that the expression of TLR4 and IL-1*β* was significantly increased in SFs separated from rat TMJ with LPS stimulation at both mRNA and protein levels, and LPS activated the TLR4 signaling pathway in SFs. However, the inflammatory response that occurred in synovial membrane is not caused by bacterial infection as we all know. In the current study, we wanted to investigate whether or not TLR4 participate in the inflammatory responses and the expression of IL-1*β* in rats induced by occlusal interference. As the results showed, the expression of TLR4 and IL-1*β* in the synovial membranes of the occlusal interference group was significantly increased at both protein and mRNA levels. This finding prompted us that maybe TLR4 participates in inflammatory response of synovial membranes in rat. So, which endogenous ligands are involved in the activation of TLR4 signaling in synovial membranes of rats in the occlusal interference group? This question remains an issue waiting for us to explore and research.

TAK-242 is a specific inhibitor of TLR4, which could selectively suppress TLR4-mediated myeloid differentiation factor 88- (MyD88-) dependent pathway as well as TIR domain-containing adapter-inducing IFN-*β* (TRIF) dependent pathway by binding to Cys747 in the intracellular domain of TLR4 and its inhibitory effect, is largely unaffected by LPS concentration and types of TLR4 ligands, and finally inhibits the expression of NO, TNF-*α*, IL-6, and IL-1*β* [28, 29]. In previous researches, TAK-242 played a protective effect in LPS-induced lung injury [30], and treatment with TAK-242 showed benefits for sepsis [31]. In the current study, we created an occlusal interference animal model by bonding crowns with a thickness of 0.6 mm to right mandibular first molars and observed inflammation changes as well as increased expression of IL-1*β* at both mRNA and protein levels. However, the effect of occlusal interference was significantly decreased by the use of TAK-242. In the TAK-242 group, the histologic severity score of synovial membranes became significantly lower after treatment with the TAK-242 when compared with the occlusal interference group. Consistent with the inflammatory reactions, the increased expression of IL-1*β* was obviously reduced at both mRNA and protein levels. These may represent an important link between activation of TLR4 and the increased expression of proinflammatory cytokines like IL-1*β* and the inflammatory reactions of synovial membranes in rats treated with occlusal interference. However, the adaptor molecules participate in intracellular signaling and the pathways of intracellular signaling transduction triggered by TLR4 and induce production of inflammatory mediators like IL-1*β*, which were not studied.

In the current study, we demonstrated that TLR4 involved in the inflammatory reactions of synovial membranes and the expression of IL-1*β* at both mRNA and protein levels caused by occlusal interference in rats. Additionally, the injection of TAK-242 could inhibit the development of this disease. The results prompted us that the activation of TLR4 may be involved in the inflammatory reactions and increased expression of IL-1*β* and participate in the mechanisms of the initiation and development of synovial injury by regulating the expression of inflammatory mediators like IL-1*β* in synovial membranes. Our research results provided new theoretical evidences for study about pathogenesis of synovitis in TMJ.
